# Draft Genome Sequence of *Eggerthia catenaformis* Strain MAR1 Isolated from Saliva of Healthy Humans

**DOI:** 10.1128/genomeA.00638-17

**Published:** 2017-07-13

**Authors:** M. Ajijur Rahman, Peter Mullany, Adam P. Roberts

**Affiliations:** a Department of Microbial Diseases, Eastman Dental Institute, University College London, London, United Kingdom; b Department of Pharmacy, University of Rajshahi, Rajshahi, Bangladesh; c Department of Parasitology, Liverpool School of Tropical Medicine, Liverpool, United Kingdom

## Abstract

Here, we report the draft genome sequence of *Eggerthia catenaformis* MAR1 isolated during a screen for d-cycloserine-resistant bacteria from the saliva of healthy humans. Analysis of the genome reveals that the strain has the potential to be a human pathogen and carries genes related to virulence and antibiotic resistance.

## GENOME ANNOUNCEMENT

*Eggerthia catenaformis* is a Gram-positive, anaerobic, non-spore-forming rod that was first isolated from human stools in 1935 (1) and later from intestinal and pleural infections (2). Based on 16S rRNA gene sequence, *E. catenaformis* was reclassified in 2011 from *Lactobacillus catenaformis* (2). Recently, *E. catenaformis* has been isolated from the blood cultures of a patient suffering from bacteremia associated with a dental abscess (3). The bacteremia caused by* E. catenaformis* was successfully treated with intravenous benzylpenicillin and metronidazole, to which the isolate was susceptible (3).

We screened for d-cycloserine-resistant anaerobic bacteria in saliva from healthy humans by plating out saliva on fastidious anaerobic agar plates supplemented with 10% fetal calf serum (FCS), 5% sheep blood, 64 µg/ml d-cycloserine, and 5 µg/ml rifampicin. Isolates were identified as *E. catenaformis* based on 16S rRNA gene sequence and phylogenetic relationships with their closest homologues. The partial 16S rRNA gene (1,358 bp) of the strain MAR1 was 99.48% identical to the *E. cateformis* DSM 20559 (ATCC 25536), the only strain of which the draft genome sequence is available in the NCBI database (GenBank accession no. AUGJ00000000).

The isolate MAR1 was selected for whole-genome shotgun sequencing. Genomic DNA was extracted from MAR1 using Gentra Puregene yeast/bact. kit (Qiagen). Genomic libraries of MAR1 were prepared using Nextera XT library prep kit (Illumina) and sequenced by MicrobesNG (https://microbesng.uk/) on the Illumina HiSeq using a 250-bp paired-end protocol. Reads were adapter trimmed using Trimmomatic version 0.30 (4), and *de novo* assembly was performed using SPAdes version 3.7 (5). The genome was annotated using RAST server version 2 (6). PathogenFinder (7) was used to estimate the pathogenicity potential of the isolate.

The draft genome comprises 621 contigs (>300 bp) with a total size of 2,358,916 bp and an average depth of coverage of 316.9×. The G+C content of the genome was calculated to be 36.1% by the RAST server, and the estimated *N*_50_ value was 110,956 bp. The draft genome contains a total of 2,298 predicted protein-coding sequences and 58 RNA-coding sequences. Twelve genes were predicted to encode proteins for multidrug-resistant efflux pumps of the resistance-nodulation-cell division (RND) and multidrug and toxic compound extrusion (MATE) family. Two putative antibiotic resistance genes that encode resistance to tetracycline [*tet*(W)] (contig 21) and aminoglycosides (*aac*) (contig 4) were found to be located within separate putative integrative mobile genetic elements. These are absent in DSM 20559. The organism was predicted to be a human pathogen, with a probability score of 0.892 by PathogenFinder.

As *E. catenaformis* has been previously isolated from several infection sites, and its genome seems primed for a pathogenic interaction with the host, it should be monitored carefully as a possible emerging human pathogen. It is envisaged that this second draft genome sequence of *E. catenaformis* will be useful in determining the potential pathogenicity as well as antimicrobial resistance capabilities of future isolates.

Ethical approval for collection of saliva from healthy volunteers was obtained from the University College London (UCL) Ethical Committee (project number 5017/001).

### Accession number(s).

This whole-genome shotgun project has been deposited at DDBJ/ENA/GenBank under the accession no. NCVR00000000. The version described in this paper is version NCVR01000000.
